# Anti-MRSA Cephalosporin versus Vancomycin-Based Treatment for Acute Bacterial Skin and Skin Structure Infection: A Systematic Review and Meta-Analysis of Randomized Controlled Trials

**DOI:** 10.3390/antibiotics10081020

**Published:** 2021-08-22

**Authors:** Ching-Yi Chen, Wang-Chun Chen, Chih-Cheng Lai, Tzu-Ping Shih, Hung-Jen Tang

**Affiliations:** 1 Division of Chest Medicine, Department of Internal Medicine, E-Da Hospital, I-Shou University, Kaohsiung 82445, Taiwan; ed109604@edah.org.tw; 2 Institute of Biotechnology and Chemical Engineering, I-Shou University, Kaohsiung 82445, Taiwan; ed101150@edah.org.tw; 3 Department of Pharmacy, E-Da Hospital, Kaohsiung 82445, Taiwan; 4 Department of Internal Medicine, Kaohsiung Veterans General Hospital, Tainan Branch, Tainan 71051, Taiwan; n261@mail.vhyk.gov.tw; 5 Department of Family Medicine, Kaohsiung Veterans General Hospital, Tainan Branch, Tainan 71051, Taiwan; 6 Department of Medicine, Chi Mei Medical Center, Tainan 71004, Taiwan

**Keywords:** acute bacterial skin and skin structure infection, ceftaroline, ceftobiprole, vancomycin, methicillin-resistant *Staphylococcus aureus*

## Abstract

This systematic review and meta-analysis of randomized controlled trials (RCTs) compared the clinical efficacy and safety of anti-MRSA cephalosporin and vancomycin-based treatment in treating acute bacterial skin and skin structure infections (ABSSSIs). PubMed, Embase, Cochrane Central Register of Controlled Trials, Turning Research into Practice, and ClinicalTrials.gov databases were searched for relevant articles from inception to 15 June 2020. RCTs comparing the clinical efficacy and safety of anti-MRSA cephalosporin with those of vancomycin-based regimens in treating adult patients with ABSSSIs were included. The primary and secondary outcomes were clinical response at the test-of-cure assessments and risk of adverse events (AEs), respectively. Eight RCTs were enrolled. The clinical response rate was not significantly different between anti-MRSA cephalosporin and vancomycin-based treatments (odds ratio [OR], 1.05; 95% CI, 0.90–1.23; *I*^2^ = 0%). Except for major cutaneous abscesses in which anti-MRSA cephalosporin-based treatment was associated with a lower clinical response rate than vancomycin-based treatment (OR, 0.62; 95% CI, 0.40–0.97; *I*^2^ = 0%), other subgroup analyses according to the type of cephalosporin (ceftaroline or ceftobiprole), type of infection, and different pathogens did not show significant differences in clinical response. Anti-MRSA cephalosporin-based treatment was only associated with a higher risk of nausea than vancomycin-based treatment (OR, 1.41; 95% CI, 1.07–1.85; *I*^2^ = 0%). In treating ABSSSIs, the clinical efficacy of anti-MRSA cephalosporin is comparable to that of vancomycin-based treatment, except in major cutaneous abscesses. In addition to nausea, anti-MRSA cephalosporin was as tolerable as vancomycin-based treatment.

## 1. Introduction

The incidence of acute bacterial skin and skin structure infections (ABSSSIs) is increasing in both community and hospital settings [1,2,3,4]. The presentation of ABSSSIs can range from mild and self-limited to more severe skin infections and may involve deeper structures, including the fascia and muscle [5,6]. Appropriate antibiotic and source control are the keys to the successful management of ABSSSIs. Methicillin-resistant *Staphylococcus aureus* (MRSA) is a principal causative pathogen of ABSSSIs in both adult and pediatric patients and has become a serious concern [7,8,9]. To treat MRSA infection, vancomycin, teicoplanin, daptomycin, and linezolid are the most commonly recommended antimicrobial agents [10].

In addition, two fifth-generation cephalosporins, ceftaroline, and ceftobiprole, which have anti-MRSA activity against common gram-negative pathogens, were developed to enrich the pharmacological armamentarium for ABSSSIs [11,12]. In vitro studies have shown that both ceftaroline and ceftobiprole exhibit potent in vitro activity against commonly encountered gram-positive bacteria, including MRSA, penicillin-resistant Streptococcus pneumoniae, Enterococcus faecalis, and gram-negative pathogens such as *Citrobacter* spp., *Escherichia coli*, *Enterobacter* spp., *Klebsiella* spp., and *Serratia* spp. [13,14,15,16]. Therefore, these two anti-MRSA cephalosporins could be recommended as therapeutic options for ABSSSIs. Recently, several randomized controlled trials (RCTs) have reported the clinical efficacy and safety of ceftaroline and ceftobiprole in the treatment of ABSSSIs [17,18,19,20,21,22,23]. However, meta-analyses comparing the use of these two anti-MRSA cephalosporins and vancomycin-based regimens against ABSSSI are lacking. Therefore, we conducted this systematic review and meta-analysis of RCTs to investigate the clinical efficacy and safety of the anti-MRSA cephalosporins, ceftaroline and ceftobiprole, for treating ABSSSIs, compared with vancomycin-based regimens.

## 2. Methods

### 2.1. Study Search and Selection

PubMed, Embase, Cochrane Central Register of Controlled Trials, Turning Research into Practice, and ClinicalTrials.gov databases were searched for relevant articles from inception to 15 June 2020. The following search terms were used: “ceftaroline”, “ceftobiprole”, “skin infection”, “complicated skin and skin structure infection”, and “acute bacterial skin and skin structure infection.” Only RCTs that compared the clinical efficacy and safety of ceftaroline or ceftobiprole with that of vancomycin-containing regimens in treating adult patients with ABSSSIs were included. The reference lists from relevant articles were manually searched for additional eligible articles. No language limitation was applied. Studies were included if they met the following criteria: (1) patients with ABSSSIs were examined; (2) anti-MRSA cephalosporin (ceftaroline or ceftobiprole) was used as an intervention; (3) the comparison included vancomycin; and (4) the study outcomes were clinical efficacy and the risk of adverse events (AEs). We excluded in vitro activity research, animal studies, and pharmacokinetic-pharmacodynamic assessments. Two investigators independently screened and reviewed each study. If any disagreement arose, a third investigator was consulted. For each included study, we extracted the following data: year of publication, study design, antimicrobial regimens, clinical outcomes, and risk of AEs. This systematic review followed the Preferred Reporting Items for Systematic Reviews and Meta-Analyses guidelines [24].

### 2.2. Outcome Measurement

The primary outcome was clinical response at the test of cure (TOC), defined as 7 days (±2 days) after the end of antibiotic treatment. Clinical response was defined as complete or near-complete resolution of baseline signs and symptoms of the primary infection with no further need for antibacterial treatment. The secondary outcome was the risk of AEs.

### 2.3. Data Analysis

The Cochrane risk-of-bias tool was used to assess the quality of the included RCTs and their associated risk of bias. Statistical analyses were performed using Review Manager (version 5.3; Nordic Cochrane Center, Copenhagen, Denmark) with the random effects model. Pooled odds ratios (ORs) and 95% CIs were calculated for outcome analyses.

## 3. Results

### 3.1. Study Selection

**Figure 1:**
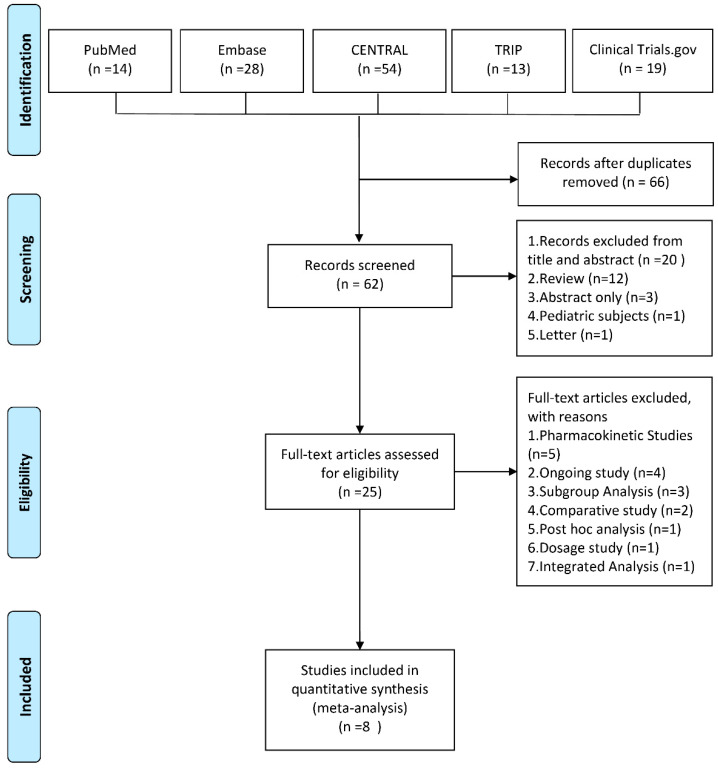
Algorithm of study selection. CENTRAL, Cochrane Central Register of Controlled Trials; TRIP, Turning Research into Practice.

The online database search initially yielded 128 articles, and 66 articles were excluded due to duplication. After screening the titles and abstracts, 37 irrelevant articles were excluded. After screening the full texts, 17 studies were excluded further. Finally, eight clinical studies [17,18,20,21,22,23,25,26] were included in the meta-analysis (Figure 1).

### 3.2. Study Characteristics

**Figure 2:**
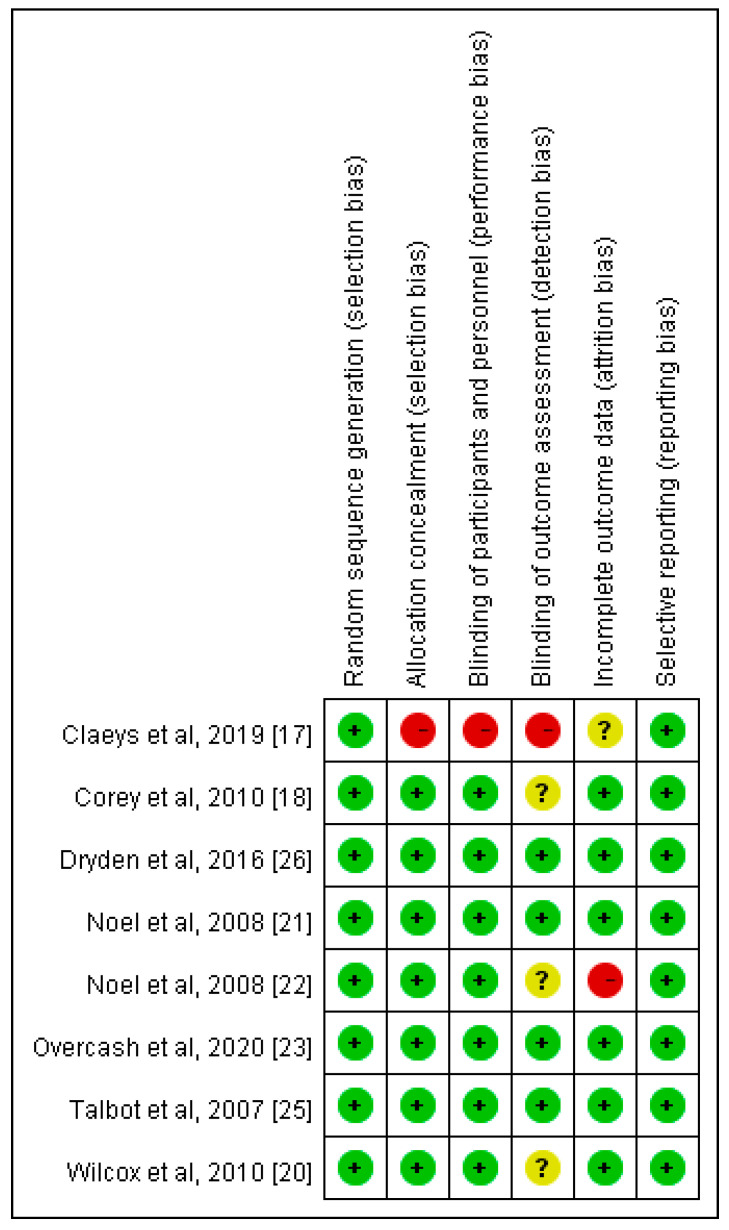
Summary of risk of bias.

Overall, eight RCTs [17,18,20,21,22,23,25,26] were included in the meta-analysis (Table 1). All were multicenter studies, and ceftaroline and ceftobiprole were assessed as experimental drugs in five and three RCTs, respectively. The comparative antibiotic regimen was vancomycin alone or plus ceftazidime or aztreonam. In summary, 2627 and 2076 patients were randomly assigned as anti-MRSA cephalosporin-based and vancomycin-based groups, respectively. Among the study group who receiving anti-MRSA cephalosporin-based treatment, 1348 and 1279 patients received ceftaroline and ceftobiprole, respectively. The assessment of the risk of bias is summarized in Figure 2. Most of the others were classified as having a low risk of bias, except for Claeys et al.’s study [17], which had a high risk of bias in selection, performance, and detection, and Noel et al. ’s study [21], which had a high risk of bias in the attrition domain.

### 3.3. Clinical Efficacy

**Figure 3:**
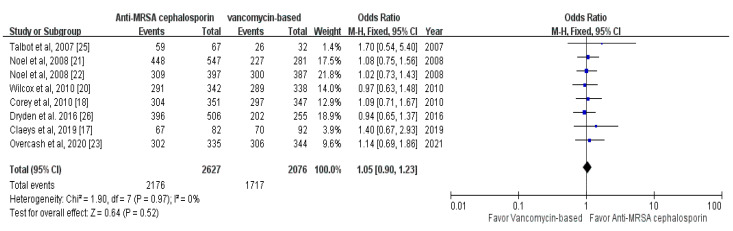
Forest plot of the comparison of clinical response rates between anti-MRSA cephalosporin-based and vancomycin-based treatments.

Overall, the clinical response at TOC was 82.8% (2176/2627) and 82.7% (1717/2076) in the study (anti-MRSA cephalosporin-based treatment) and control (vancomycin-based treatment) groups, respectively. Furthermore, no significant difference in the clinical response rate at the TOC visits was observed between the study and control groups (OR, 1.05; 95% CI, 0.90–1.23; *I*^2^ = 0%; Figure 3). The similarity in the clinical response between the anti-MRSA cephalosporin-based treatment and vancomycin-based treatment remained unchanged in the sensitivity test in which individual studies were randomly excluded. In a subgroup analysis, no significant difference was observed in the clinical response between ceftaroline or ceftobiprole and vancomycin or linezolid (ceftaroline: 82.9% [1117/1348] vs. 83.1% [884/1064]; OR, 1.04; 95% CI, 0.84–1.30; *I*^2^ = 0%; ceftobiprole: 82.8% [1059/1279] vs. 82.3% [833/1012]; OR, 1.06; 95% CI, 0.85–1.33; *I*^2^ = 0%).

A subgroup analysis according to each pathogen showed no significant difference in the clinical response between anti-MRSA cephalosporin-based treatment and vancomycin-based treatment in patients with *S. aureus* (92.8% [794/856] vs. 90.9% [641/705]; OR, 1.25; 95% CI, 0.87–1.82; *I*^2^ = 0%), methicillin-sensitive *S. aureus* (MSSA) (93.4% [696/745] vs. 91.2% [577/633]; OR, 1.33; 95% CI, 0.89–1.99; *I*^2^ = 2%), MRSA (91.3% [376/412] vs. 91.3% [284/311]; OR, 1.04; 95% CI, 0.62–1.76; *I*^2^ = 0%), *Streptococcus pyogenes* (95.8% [114/119] vs. 95.1% [97/102]; OR, 1.29; 95% CI, 0.39–4.25; *I*^2^ = 0%), *Streptococcus agalactiae* (93.1% [27/29] vs. 89.7% [26/29]; OR, 1.08; 95% CI, 0.20–5.93, *I*^2^ = 0%), and *Streptococcus anginosus* (90.0% [9/10] vs. 81.8% [9/11]; OR, 2.50; 95% CI, 0.16–38.60) infections.

Further subgroup analysis according to the type of infection was conducted to assess the clinical response between anti-MRSA cephalosporin-based and vancomycin-based treatments in patients with cellulitis, wound infection, and major cutaneous abscess. No significant difference in the clinical response between anti-MRSA cephalosporin-based and vancomycin-based treatments in patients with cellulitis (85.4% [643/753] vs. 85.3% [473/554]; OR, 1.22; 95% CI, 0.88–1.68; *I*^2^ = 0%), and wound infection (89.0% [413/464] vs. 89.7% [350/392]; OR, 0.96; 95% CI, 0.62–1.49; *I*^2^ = 0%). In contrast, anti-MRSA cephalosporin-based treatment was associated with a lower clinical response rate in patients with major cutaneous abscess than vancomycin-based treatment (90.5% [631/697] vs. 94.2% [552/554]; OR, 0.62; 95% CI, 0.40–0.97; *I*^2^ = 0%).

### 3.4. Adverse Events

Overall, no significant difference between anti-MRSA cephalosporin-based treatment and vancomycin-based treatment was observed in terms of the risk of treatment-emergent AE (OR 0.96, 95% CI 0.85–1.09, *I*^2^ = 26%), serious AEs (OR 0.90, 95% CI 0.67–1.22, *I*^2^ = 0%), AEs leading to treatment discontinuation (OR 0.74, 95% CI 0.53–1.04, *I*^2^ = 0%), and death (OR 1.82, 95% CI 0.38–8.57, *I*^2^ = 46%).

**Figure 4:**
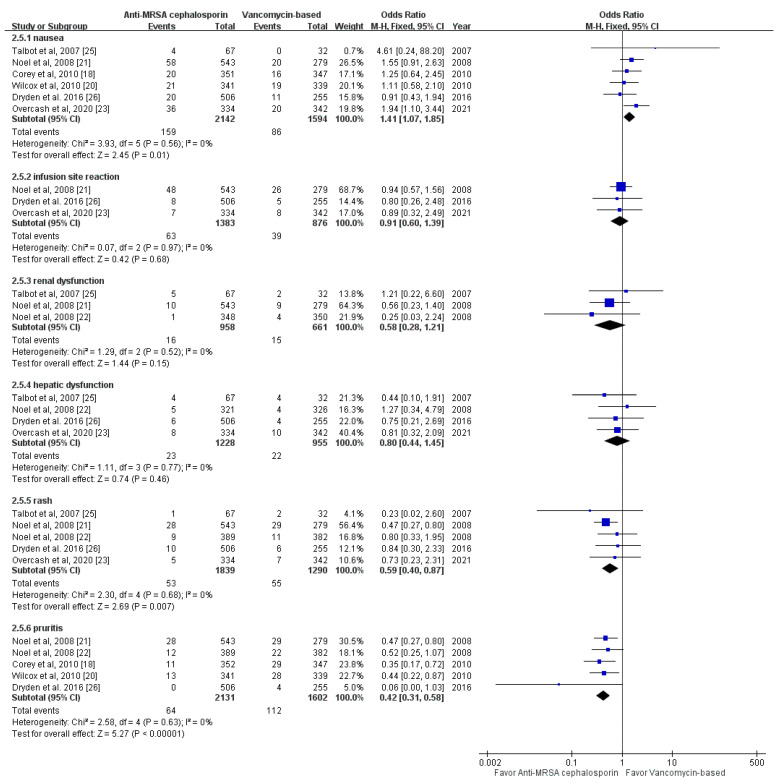
Forest plot of the comparison of the risk of specific adverse events between anti-MRSA cephalosporin-based and vancomycin-based treatments.

For specific AEs, anti-MRSA cephalosporin-based treatment was associated with a higher risk of nausea than vancomycin-based treatment (OR, 1.41; 95% CI, 1.07–1.85; *I*^2^ = 0%; Figure 4). In contrast, anti-MRSA cephalosporin-based treatment was associated with a lower risk of rash and pruritis than vancomycin-based treatment (rash: OR, 0.59; 95% CI, 0.40–0.87; *I*^2^ = 0%; pruritis: OR, 0.42; 95% CI, 0.31–0.58; *I*^2^ = 0%). Otherwise, no significant difference between anti-MRSA cephalosporin-based treatment and vancomycin-based treatment was observed in terms of the risk of renal dysfunction (OR, 0.58; 95% CI, 0.28–1.21; *I*^2^ = 0%), infusion site reaction (OR, 0.91; 95% CI, 0.60–1.392; *I*^2^ = 0%), and abnormal liver function (OR, 0.80; 95% CI, 0.44–1.45; *I*^2^ = 0%) (Figure 4).

## 4. Discussion

This meta-analysis included eight RCTs to compare the efficacy of anti-MRSA cephalosporins and vancomycin-based regimens in patients with ABSSSIs. We found that anti-MRSA cephalosporins were at par with the comparators in ABSSSI treatment, and this finding was supported by the following evidence. First, with respect to the clinical response among patients with ABSSSIs, anti-MRSA cephalosporin-based treatments were comparable to vancomycin-based treatments, and the leave-one-out sensitivity analysis did not change the results. Second, this similarity remained unchanged in the subgroup analyses according to ceftaroline and ceftobiprole. Third, anti-MRSA cephalosporins were comparable to vancomycin in the treatment of ABSSSIs in all subgroup analyses according to different causative pathogens, including MRSA. Finally, based on the findings of the subgroup analysis according to the type of ABSSSIs, we found no significant difference in the clinical response between anti-MRSA cephalosporins and vancomycin-based regimens in the treatment of cellulitis and wound infection. Overall, our findings were consistent with those of previous meta-analyses [27,28] of RCTs according to ceftaroline and ceftobiprole, respectively, and provided additional evidence supporting the usefulness of anti-MRSA cephalosporin in the treatment of ABSSSIs. Moreover, this study demonstrated that the clinical efficacy of anti-MRSA cephalosporin-based treatment was comparable to vancomycin-based treatment and suggested that anti-MRSA cephalosporin could be an alternative antibiotic of choice to spare the use of vancomycin.

In contrast to the above findings, we found one exception: major cutaneous abscess, in which anti-MRSA cephalosporin-based treatments were associated with a lower clinical response rate than vancomycin-based treatment (OR, 0.62; 95% CI, 0.40–0.97; *I*^2^ = 0%). However, the clinical response rate in this setting remained favorable, achieving 90.5% in the present study. Further analysis showed that the clinical response rate was numerically lower in ceftaroline or ceftobiprole compared to vancomycin-based treatment, but the difference did not reach statistical significance (ceftaroline: OR, 0.73; 95% CI, 0.41–1.29; *I*^2^ = 0%; ceftobiprole: OR, 0.49; 95% CI, 0.24–1.02; *I*^2^ = 0%). Several issues such as the inoculum effect or surgical intervention may affect the clinical outcomes of patients with abscess; however, these data was not available in this meta-analysis. Further studies are warranted to investigate the usefulness of anti-MRSA cephalosporins in the treatment of major cutaneous abscesses.

Regarding safety issues, we found that ant-MRSA cephalosporin was only associated with a higher risk of nausea than vancomycin-based treatment. Further analysis showed that this finding was mainly driven by ceftobiprole, which exhibited a significantly higher risk (OR, 1.72; 95% CI, 1.16–2.53; *I*^2^ = 0%). In contrast, ceftaroline had a similar risk of nausea to the comparator (OR, 1.14; 95% CI, 0.77–1.68; *I*^2^ = 0%). Besides nausea, anti-MRSA cephalosporin was not associated with a higher risk of AEs than vancomycin-based treatment. Dermatological AEs (i.e., rash and pruritis) were lower in the anti-MRSA cephalosporin group than in the vancomycin group. Although we found anti-MRSA cephalosporin was associated with numerically lower risk of renal and hepatic dysfunction than vancomycin-based treatment, these differences did not reach statistical significance. The cause could be due to the fact that our findings was based on the analysis of only three and five studies which reported these respective outcomes. Further study is needed to clarify these issues, particular for the risk of renal dysfunction, which may be a clear advantage of anti-MRSA cephalosporins over vancomycin.

This meta-analysis had several limitations. First, we did not assess the microbiological response or the association between antibiotic-resistant organisms and their related clinical responses. Second, anti-MRSA cephalosporin exhibits potent in vitro activity against commonly encountered gram-negative pathogens, which may be involved in both mono- and poly-microbial ABSSSIs. However, this additional effect of ceftaroline or ceftobiprole on ABSSSI caused by gram-negative pathogens was not evaluated in this study.

In conclusion, the efficacy of anti-MRSA cephalosporin-based treatment is comparable to that of vancomycin-based in treating ABSSSIs, except in major cutaneous abscesses. In addition to nausea, anti-MRSA cephalosporin was as tolerable as a vancomycin-based treatment.

## Figures and Tables

**Table 1:** Characteristics of the included studies.

Author, Year	Study Design	Study Sites	Inclusion Criteria	Study Drug	Comparator	No of ITT Population		Primary Outcome
						Study Drug	Comparator	
Talbot et al., 2007 [25]	Randomized, observer-blinded, phase 2 trial	15 sites in the US, South America, South Africa, and Russia	Adults with cSSSI requiring hospitalization and intravenous antibiotic	Ceftaroline	Vancomycin with or without aztreonam	67	32	Clinical cure rate at TOC
Claeys et al., 2019 [17]	Prospective, open-label, randomized trial	3 centers in the US	Adult patients with ABSSSI required intravenous antibiotic and at risk for MRSA	Ceftaroline	Vancomycin	82	92	Early clinical response rate
Dryden et al. 2016 [26]	Prospective, randomized, double-blind trial	111 centers in 28 countries	Adults with cSSSI requiring hospitalization and intravenous antibiotic	Ceftaroline	Vancomycin plus aztreonam	514	258	Clinical cure rate at TOC
Corey et al., 2010 [18]	Randomized, double-blind, active-controlled, parallel group trial	55 centers in 10 countries	Adults with cSSSI requiring hospitalization and intravenous antibiotic	Ceftaroline	Vancomycin plus aztreonam	353	349	Clinical cure rate at TOC
Wilcox et al., 2010 [20]	Randomized, double-blind, active-controlled, parallel group trial	56 centers in 12 countries	Adults with cSSSI requiring intravenous antibiotic	Ceftaroline	Vancomycin plus aztreonam	348	346	Clinical cure rate at TOC
Noel et al., 2008 [21]	Randomized, double-blind trial	129 sites in North America, Europe, South America, Asia, and Africa	Adults with cSSSI requiring intravenous antibiotic	Ceftobiprole	Vancomycin plus ceftazidime	547	281	Clinical and microbiological outcomes at TOC
Noel et al., 2008 [22]	Randomized, double-blind trial	129 sites in Europe, Asia, Africa, South America, and North America	Adults with cSSSI due to documented or suspected gram-positive pathogen	Ceftobiprole	Vancomycin	397	387	Clinical cure rate at TOC
Overcash et al., 2020 [23]	Randomized, double-blind, active-controlled, parallel-group trial	32 sites in the US, Bulgaria, Hungary, and Ukraine	Adult patients with ABSSSI required hospitalization and intravenous antibiotic	Ceftobiprole	Vancomycin plus aztreonam	335	344	Early clinical response and clinical success at TOC

TOC, test of cure; cSSSI, complicated skin and skin structure infection; ABSSSI, acute bacterial skin and skin structure infection; MRSA, methicillin-resistant Staphylococcus aureus; ITT, intention-to-treat.
